# The Role of Gene Conversion between Transposable Elements in Rewiring Regulatory Networks

**DOI:** 10.1093/gbe/evz124

**Published:** 2019-06-18

**Authors:** Jeffrey A Fawcett, Hideki Innan

**Affiliations:** 1 RIKEN iTHEMS, Wako, Saitama, Japan; 2 SOKENDAI, Hayama, Kanagawa, Japan

**Keywords:** transposable elements, rewiring regulatory network, gene conversion

## Abstract

Nature has found many ways to utilize transposable elements (TEs) throughout evolution. Many molecular and cellular processes depend on DNA-binding proteins recognizing hundreds or thousands of similar DNA motifs dispersed throughout the genome that are often provided by TEs. It has been suggested that TEs play an important role in the evolution of such systems, in particular, the rewiring of gene regulatory networks. One mechanism that can further enhance the rewiring of regulatory networks is nonallelic gene conversion between copies of TEs. Here, we will first review evidence for nonallelic gene conversion in TEs. Then, we will illustrate the benefits nonallelic gene conversion provides in rewiring regulatory networks. For instance, nonallelic gene conversion between TE copies offers an alternative mechanism to spread beneficial mutations that improve the network, it allows multiple mutations to be combined and transferred together, and it allows natural selection to work efficiently in spreading beneficial mutations and removing disadvantageous mutations. Future studies examining the role of nonallelic gene conversion in the evolution of TEs should help us to better understand how TEs have contributed to evolution.

## Evolution of Regulatory Networks

**Fig. 1.**
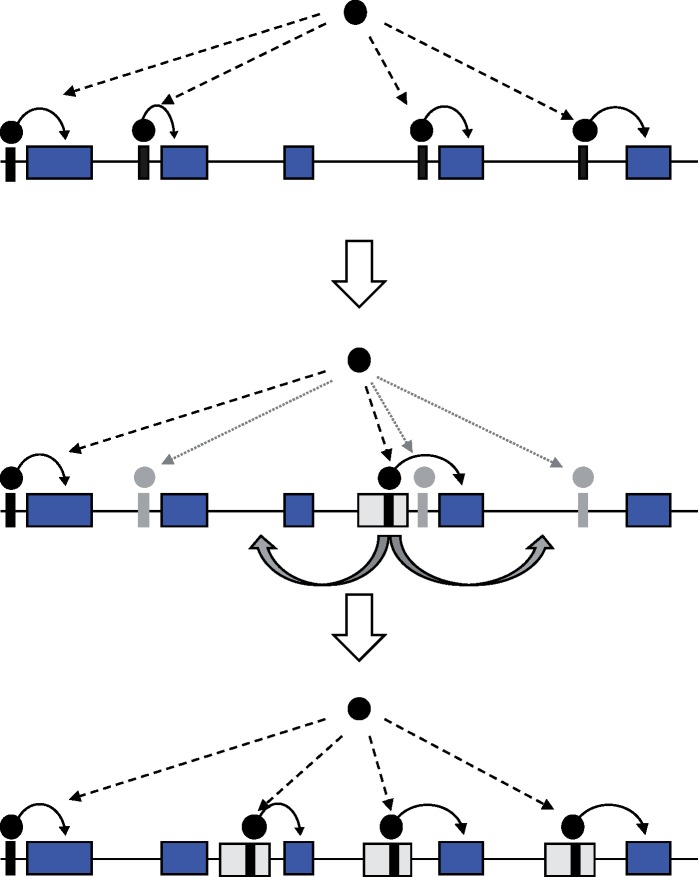
—An example of the rewiring of a gene regulatory network where a DNA-binding protein (black circles) regulates a number of genes (blue rectangles) by binding to DNA motifs (black stripes). Some of the motifs and binding events may be lost (represented by gray stripes and circles), whereas new motifs and binding events may appear, which can sometimes wire new genes into the network. TEs (gray rectangles) may play an important role in providing and dispersing these new motifs.

Eukaryotic genomes contain many DNA-binding proteins which bind to thousands of sites in the genome sharing a common DNA motif. This enables the coordinated regulation of various molecular and cellular processes. For instance, there are many gene regulatory networks controlled by transcription factors that bind to promoter motifs. Other examples include PRDM9 that regulates recombination in humans and some other mammalian species (Ponting 2011), or CTCF, a DNA-binding protein responsible for the regulation of the chromatin structure (Phillips and Corces 2009). Some motifs are ∼10 bp whereas others, such as the CTCF-binding motif (Schmidt et al. 2012), are as long as ∼30 bp and most motifs allow a certain amount of mismatches. How these networks can evolve has been of great interest because 1) the coevolution involving the DNA-binding protein and so many different motifs should be extremely difficult by independent mutations, and 2) the creation of such a large number of new motifs by independent mutations should not be so easy either. Nevertheless, these networks can be quite different across species such that the binding events are often not conserved across the orthologous loci of even closely related species. Many sets of motifs appear to be subject to a high birth-and-death rate, providing ample opportunities for new genes to be wired in to the network (fig. 1) (Borneman et al. 2007; Schmidt et al. 2010).

The possible role of transposable elements (TEs) in the rewiring of regulatory networks, as illustrated in figure 1, has been discussed on several occasions (Britten and Davidson 1969; Feschotte 2008; Chuong et al. 2017). Indeed, several recent studies have shown that a significant portion of the motifs in these networks are provided by TEs (Bourque et al. 2008; Schmidt et al. 2012; Ellison and Bachtrog 2019). For instance, one study showed that up to 25% of the binding sites of CTCF, NANOG, and OCT4 in human and mouse are embedded in TEs (Kunarso et al. 2010). Many of the PRDM9 motifs in primates are provided by a number of TE families, in particular an inactive *THE1* retrotransposon (Myers et al. 2008; McVean 2010; Auton et al. 2012). One recent study reported that 178 of the 512 transcription factors tested bound to the L1 elements in humans in at least one biological condition (Sun et al. 2018), suggesting that TEs can provide the raw material for the evolution of regulatory networks. TE-mediated rewiring by-passes the difficulties associated with both the large scale coevolution and the independent creation of a large number of new identical motifs. This is because TEs can provide a large number of highly similar motifs dispersed throughout the genome that are ready-to-use, and disperse the motif to a large number of genomic loci within a relatively short period of time. Especially for the networks involving longer motifs (e.g., CTCF), the contribution of TEs might have been crucial because they hardly arise by chance. Here, we will argue that the rewiring process can be even more effective when nonallelic gene conversion is occurring between the TE copies. For instance, many of the binding efficiency of the motifs provided by TEs might be initially suboptimal that can be further improved by mutations. Nonallelic gene conversion allows such beneficial mutations to be shared across the different TE copies instead of the whole rewiring process by transposition having to take place each time a better motif appears in one of the copies. Below, we will first review evidence of nonallelic gene conversion in the evolution of TEs. Then, we will demonstrate the advantages of nonallelic gene conversion and discuss its role in rewiring gene regulatory networks. Note that although we will use the term “gene regulatory networks,” the discussion should apply to any process that requires the recognition of many near-identical motifs dispersed throughout the genome, such as those mediated by PRDM9 or CTCF.

## Nonallelic Gene Conversion in TEs

**Fig. 2.**
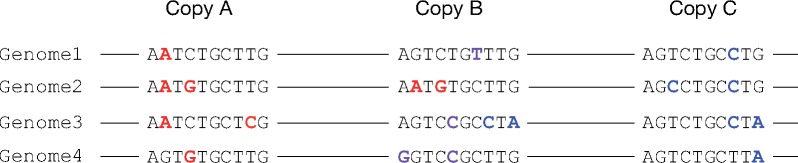
—Pattern of polymorphism observed with nonallelic gene conversion. Nucleotide polymorphism of four individual genomes for three TE copies is shown. Nucleotides in red, purple, blue are polymorphisms due to point mutations in Copies A, B, and C, respectively. The mutations in Copy A in Genome 2 are transferred to Copy B, whereas the mutations in Copy C in Genome 3 are transferred to copy B by nonallelic gene conversions.

Nonallelic gene conversion occurs between highly similar homologous sequences such as duplicated sequences or TEs (Chen et al. 2007; Fawcett and Innan 2011). Nonallelic gene conversion can transfer a new mutation from one copy to the other copy, or reverse the mutation to its original state. Because of this, copies undergoing nonallelic gene conversion will remain highly similar to each other. The significance of nonallelic gene conversion in the evolution of multigene families has been well studied and some studies have also reported nonallelic gene conversion in TEs. Theoretically, one consequence of gene conversion is that the level of polymorphism within each copy increases because of the sharing of mutations (Innan 2002, 2003). In such cases, a number of “shared” polymorphic sites, where the same polymorphic nucleotides are present in both copies, are typically observed. When multiple copies are involved, a complex mosaic pattern of polymorphism is typically observed as shown in figure 2 where a particular region within a copy of some individuals are identical to another copy, whereas a different region within the copy is identical to yet another different copy. This was reported for LTR retrotransposons on human Y chromosomes (Trombetta et al. 2016). The authors studied the pattern of polymorphisms in 52 LTRs and found that some LTRs had much higher nucleotide diversity compared with their flanking regions, one LTR in particular showing higher nucleotide diversity than the most diverse ectopic gene conversion hotspot on the Y chromosome. Within these LTRs, it was observed that the same samples contained clusters of multiple derived alleles that are located close to each other, suggesting that these SNPs were transferred by a gene conversion event of this region from another LTR elsewhere in the genome. Indeed, such LTRs with corresponding sequences that probably acted as the gene conversion “donor” were also identified. Note that some TEs such as LTR retrotransposons contain repeats such as LTRs within the same copy and nonallelic gene conversion can occur between these repeats of the same copy (Kijima and Innan 2010), although we will not discuss these cases in this article.

Another consequence of nonallelic gene conversion is reduced divergence between copies. Without nonallelic gene conversion, each copy should accumulate mutations independently, thereby increasing the divergence between copies almost linearly. By contrast, with frequent nonallelic gene conversion, the divergence does not increase linearly and instead stays around an equilibrium for a long time (Teshima and Innan 2004). This state is known as concerted evolution, during which copies undergo coevolution. Concerted evolution causes an incongruence between the real history and observed gene tree. Many studies have reported such incongruences in *Alu*, a SINE retrotransposon that is the most abundant TE family in the human genome. *Alu* elements are classified into a number of subfamilies corresponding to their insertion ages based on a number of “diagnostic mutations” (Batzer and Deininger 2002). Nonallelic gene conversion in *Alu* has been documented based on careful analysis of the pattern of these diagnostic mutations. Some copies show mosaic patterns of diagnostic mutations representative of different subfamilies, while in other cases, copies from different subfamilies occupy the same orthologous position in different primate species (Kass et al. 1995; Roy et al. 2000; Salem et al. 2005; Styles and Brookfield 2009). For instance, one early study reported a locus in human which was occupied by a young and mostly human-specific *Alu* subfamily. However, the orthologous loci in chimpanzee, gorilla, orangutan, and green monkey all contained a highly mutated *Alu* element belonging to an old subfamily, suggesting that the element was replaced by a nonallelic gene conversion event in the human lineage by an element belonging to a young subfamily (Kass et al. 1995). The general pattern of nonallelic gene conversion in *Alu* appears to be similar to that in duplicated genes or segmental duplications. The rate of nonallelic gene conversion is related to the distance and identity between the copies, as reported for duplicated genes (Mansai et al. 2011), with elevated rates of gene conversion observed between copies within <10 kb and also at certain hotspots, especially on the sex chromosomes (Aleshin and Zhi 2010).

It has been reported for the Ty retrotransposon in *Saccharomyces cerevisiae* and the Tf2 retrotransposon in *Schizosaccharomyces pombe* that nonallelic gene conversion can sometimes occur by homologous recombination between the reverse-transcribed cDNA and the chromosomal copies of these TE elements (Melamed et al. 1992; Hoff et al. 1998). The case of Tf2 is particularly interesting because it seems that their ability to produce “true” transpositions is very low, although they are capable of “mobilizing” by replacing existing copies through homologous recombination with their cDNA (Hoff et al. 1998). Many highly similar Tf2 elements exist in the *S. pombe* genome (Bowen et al. 2003). It may be that these Tf2 elements were initially propagated by the transpositional machinery of the closely related Tf1 retrotransposon in *trans* and that they have since been maintaining high-sequence similarity by frequent nonallelic gene conversion via cDNA intermediates (Hoff et al. 1998). It remains to be investigated how common this mechanism of nonallelic gene conversion is.

## Advantages of Nonallelic Gene Conversion in Rewiring Networks

**Fig. 3.**
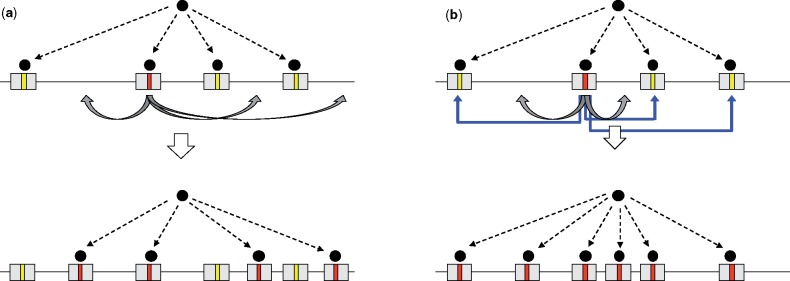
—Rewiring of a regulatory network mediated by TEs without nonalleic gene conversion (*a*) and with nonallelic gene conversion (*b*). Initially, a DNA-binding protein (black circle) binds to a particular motif (yellow stripes) contained within a given TE family (gray rectangles). A new motif (red stripe) that confers an advantage to the system (e.g., improves the binding efficiency) arises by mutation to the initial motif. This new motif can be preferentially utilized by being dispersed to several new genomic locations by transposition of the TE copy (gray arrows) containing the motif (*a*), or by being dispersed by transposition and also by replacing the old motif with the new motif via nonallelic gene conversion (blue arrows) (*b*).

Nonallelic gene conversion between the TE copies may provide huge advantages in rewiring gene regulatory networks, as illustrated in figure 3. This was recently demonstrated for the dosage compensation system of the *Drosophila miranda* sex chromosome (Ellison and Bachtrog 2015). In the recently established neo-X chromosome of *D. miranda*, dosage compensation is achieved by a ribonucleoprotein complex binding to a specific motif that was dispersed across the neo-X chromosome as part of a Helitron family called ISX (Ellison and Bachtrog 2013). ISX contains a 10-bp deletion compared with the more abundant ISY element and this 10-bp deletion resulted in the creation of the functional binding motif. Interestingly, the authors showed that the binding affinity of this motif that was initially dispersed was suboptimal. The currently dominant motif has two mutations separated by two base pairs, and has a higher binding affinity than the original motif. It seems likely that the haplotype containing the two mutations first arose in an ISY element, which does not contain a functional motif due to the lack of the 10-bp deletion, and was transferred from to an ISX element by nonallelic gene conversion. Moreover, this motif is currently spreading across the ISX elements by gene conversion between ISX elements. It is also interesting to point out that there is epistatic interaction between the two mutations, that is, each mutation decreases the binding affinity on their own, but increases the binding affinity when they are together.

This study illustrates several advantages of nonallelic gene conversion in rewiring networks (Fawcett and Innan 2015). One obvious advantage of nonallelic gene conversion in rewiring networks is that it offers an alternative mechanism to spread beneficial mutations in addition to transposition, which could be highly beneficial because a high rate of transposition could be detrimental to the genome. Likewise, deleterious mutations can be reverted to its original state by gene conversion. Another important advantage is that selection can work much more efficiently to spread beneficial mutations and eliminate deleterious mutations when nonallelic gene conversion is occurring between the different copies, which has been demonstrated for multigene families (Mano and Innan 2008). Indeed, selection was found to be playing an important role in spreading the beneficial mutations across the ISX elements (Ellison and Bachtrog 2015). This advantage of nonallelic gene conversion can be described theoretically as follows. Consider a Wright-Fisher population with *N* diploids, where each haploid individual has *n* loci (copies) that are similar enough to each other so that nonallelic gene conversion can occur between them. In this mode, it can be considered that each of the *n* loci constitutes a gene pool of 2 *N* haploid individuals. Suppose that a new mutation arises in one locus in one individual. When *s* is the selection coefficient that the mutation confers (additive selection is assumed), the probability (*f*(*s*)) that this new mutation spreads and fixes in the entire gene pool of 2*Nn* is:

$$f(s)=\frac{1-e^{-2s}}{1-e^{-2n\mathrm{Ns}}}$$

Then, because the expected number of mutations in the entire gene pool per generation is 2Nnμ, the evolutionary rate (the substitution rate) is given by:

F(s)=2Nnμf(s).

**Fig. 4.**
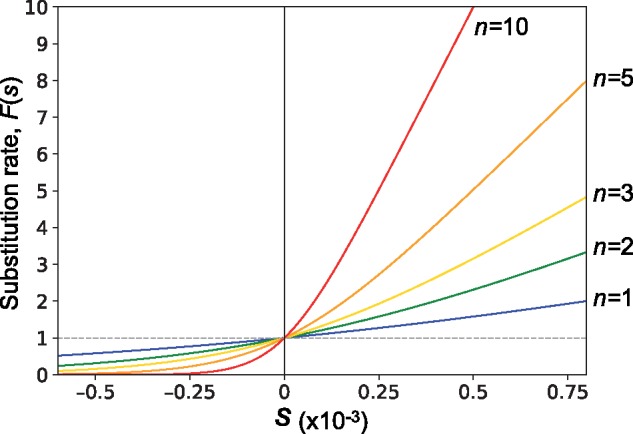
—The substitution rate (*F*(*s*)) as a function of *s*, the selection coefficient that a single mutation confers. *N *=* *1,000 was assumed for computing *F*(*s*). *F*(*s*) is standardized such that F(0)=1.

Obviously, f(0)=1/(2Nn) for a neutral mutation, and because the expected number of mutations in the entire gene pool per generation is 2Nnμ, the evolutionary rate (the substitution rate) is given by F(0)=μ, which is identical to the prediction of Kimura’s neutral theory for a single locus system. It is important to note that *F*(*s*) is independent of the copy number *n* in a neutral case, that is, *F*(0), although *n* has a significant impact on *F*(*s*) when selection works. Briefly, as *n* increases, selection works more efficiently. It is also interesting to note that the fixation probability is independent of the gene conversion rate, although the time to fixation becomes shorter when the gene conversion rate increases (see Mano and Innan 2008). Figure 4 shows the substitution rate (*F*(*s*)) as a function of *s*, where *F*(*s*) is standardized such that F(0)=1 (*N *=* *1,000 is assumed). For a positively selected mutation with *s *>* *0, *F*(*s*) increases as *n* increases, whereas *F*(*s*) decreases for deleterious mutations (*s *<* *0). This means that, in a large family, beneficial mutations are more likely to fix, while deleterious mutations are eliminated more efficiently. Strictly speaking, this theory describes a situation where the *n* copies in the genome are stable over time (i.e., no duplication/deletion and no relocation), whereas TE copies frequently undergo turnover such that some copies generate new identical copies whereas other copies are lost. In other words, an advantageous mutation could spread throughout the genome without nonallelic gene conversion simply by amplification (transposition) of the copy containing the mutation, as long as the transposition rate of that copy (and its descendant copies) is high enough (fig. 3*a*). Nevertheless, the main point of this theory is that the fate of a mutation is determined as if the entire gene pool is treated as a single population when nonallelic gene conversion is occurring, which should also apply even when the copy number of the TE is changing (fig. 3*b*). Thus, nonallelic gene conversion provides tremendous benefits by accelerating adaptive substitutions and efficiently eliminating deleterious mutations.

Another advantage of nonallelic gene conversion is that multiple mutations located close to each other can be transferred together. Moreover, multiple mutations that occur on different backgrounds or lineages can be combined and transferred together. Importantly, some mutations should enhance or disrupt the activity of TEs (i.e., the ability to generate new copies). Because these mutations are likely to be independent of mutations that affect the binding motif, they are likely to interfere with each other without nonallelic gene conversion. If so, the fate of a mutation will depend on the activity of the copy in which they occur. Nonallelic gene conversion would allow mutations that improve the binding efficiency and mutations that enhance the spreading of the TEs to be combined and selected for efficiently. This also means that nonfunctional motifs can act as reservoirs of mutations and contribute to the gene pool, as suggested with the ISY elements (Ellison and Bachtrog 2015), as long as they can engage in nonallelic gene conversion. A similar role for pseudogenes in multigene families undergoing nonallelic gene conversion has been previously demonstrated (Takuno et al. 2008).

## Advantages of Nonallelic Gene Conversion for TE Evolution

Here, we have argued that nonallelic gene conversion between TE copies provides several advantages in rewiring gene regulatory networks. The role of nonallelic gene conversion may be analogous to that of sexual recombination in that mutations on different haplotypes can be combined and selection can work more efficiently. Although we described the impact of nonallelic gene conversion between TE copies above in the context of the rewiring of networks, the same discussion should also apply to the evolution of TE families in general. Nonallelic gene conversion is likely to enhance the ability of TEs to amplify within the genome, for instance, by efficiently combining and spreading mutations that increase the transposition rate.

Currently, studies on nonallelic gene conversion in TEs are rather limited and our understanding of their effect on the evolution of TEs is rather poor. Although it is unrealistic to assume that each copy of a given TE family engage in a high rate of nonallelic gene conversion with all the other copies that are dispersed across several different chromosomes, it should be reasonable to assume that at least some of the copies located close to each other do occasionally undergo nonallelic gene conversion. Nevertheless, as we discussed earlier, even a low rate of nonallelic gene conversion should confer an advantage to the TE family. In this respect, it is worth noting that the rate of nonallelic gene conversion can be quite different across the genome. Many hotspots of nonallelic gene conversion between homologous sequences including some TEs have been identified in the human genome (Fawcett and Innan 2013). In addition, certain TEs may increase the rate of both allelic recombination and nonallelic gene conversion, and in some cases, the rate of nonallelic gene conversion by homologous recombination with cDNA as for the Tf2 retrotransposon in *S. pombe* (Hoff et al. 1998; Myers et al. 2008; Zaratiegui 2017). Thus, the presence of certain copies in genomic hotspots of nonallelic gene conversion, or the presence of certain motifs that increase the nonallelic gene conversion rate may well affect whether the TE family can successfully colonize the genome.

## Concluding Remarks

Despite being selfish genomic parasites by nature, TEs have played a crucial role in evolution by providing the raw genetic material for selection to work on. In particular, it seems that natural selection has effectively utilized TEs to form complex molecular and cellular processes that depend on the presence of hundreds or thousands of near-identical sequences across the genome (Britten and Davidson 1969; Feschotte 2008; Chuong et al. 2017). Here, we have argued that nonallelic gene conversion enhances the genetic diversity created by TEs and allows selection to utilize TEs more efficiently. Thus, further understanding of the role of nonallelic gene conversion in the evolution of TEs should enable us to better understand how TEs have contributed to evolution.
